# Early Root Overproduction Not Triggered by Nutrients Decisive for Competitive Success Belowground

**DOI:** 10.1371/journal.pone.0055805

**Published:** 2013-01-31

**Authors:** Francisco M. Padilla, Liesje Mommer, Hannie de Caluwe, Annemiek E. Smit-Tiekstra, Cornelis A. M. Wagemaker, N. Joop Ouborg, Hans de Kroon

**Affiliations:** 1 Experimental Plant Ecology, Institute for Water and Wetland Research, Radboud University Nijmegen, Nijmegen, The Netherlands; 2 Nature Conservation and Plant Ecology, Wageningen University, Wageningen, The Netherlands; 3 Molecular Ecology, Institute for Water and Wetland Research, Radboud University Nijmegen, Nijmegen, The Netherlands; University of Utah, United States of America

## Abstract

**Background:**

Theory predicts that plant species win competition for a shared resource by more quickly preempting the resource in hotspots and by depleting resource levels to lower concentrations than its competitors. Competition in natural grasslands largely occurs belowground, but information regarding root interactions is limited, as molecular methods quantifying species abundance belowground have only recently become available.

**Principal Findings:**

In monoculture, the grass *Festuca rubra* had higher root densities and a faster rate of soil nitrate depletion than *Plantago lanceolata*, projecting the first as a better competitor for nutrients. However, *Festuca* lost in competition with *Plantago*. *Plantago* not only replaced the lower root mass of its competitor, but strongly overproduced roots: with only half of the plants in mixture than in monoculture, *Plantago* root densities in mixture were similar or higher than those in its monocultures. These responses occurred equally in a nutrient-rich and nutrient-poor soil layer, and commenced immediately at the start of the experiment when root densities were still low and soil nutrient concentrations high.

**Conclusions/Significance:**

Our results suggest that species may achieve competitive superiority for nutrients by root growth stimulation prior to nutrient depletion, induced by the presence of a competitor species, rather than by a better ability to compete for nutrients per se. The root overproduction by which interspecific neighbors are suppressed independent of nutrient acquisition is consistent with predictions from game theory. Our results emphasize that root competition may be driven by other mechanisms than is currently assumed. The long-term consequences of these mechanisms for community dynamics are discussed.

## Introduction

Co-occurring plant species frequently share space and compete belowground for essential soil nutrients [1], [2]. Competition theory predicts that plant species win competition for a shared resource by more quickly preempting the resource supply in hotspots, as a result of greater root plasticity [1], [3]–[5], and by depleting resource levels to lower concentrations than their competitors [6]–[8]. In competition studies with two species, the winner takes the share of the inferior species if resource availability is finite. This results in a competitive replacement where the superior species grows at the expense of the inferior [1], [9]–[11], with the total aboveground yield of the mixture being intermediate to that of the monocultures [9]–[11]. Mixtures can draw more resources and will produce more biomass than the average of the monocultures (“overyield”) if species occupy different niches, such as different rooting depths, take up different nutrient sources, or if they segregate in phenology [10], [12]–[19].

This classical model of resource competition and plasticity to nutrients does not take into account responses to neighbors independent of responses to nutrients [20], [21]. Game theory predicts that plants should allocate a much greater share of their resources to roots than in the absence of competition, in order to prevent competitors from capturing the nutrients [22]. Evidence from pot experiments with individual plants is accumulating that such responses exist, independent of nutrient acquisition [23]–[27], but to what extent they affect the competition between plant populations of different species has not been examined so far.

Testing these predictions requires that root investments of different species are quantified in mixtures but such information is rarely available [28], as molecular methods quantifying species abundance belowground have only recently become available [29], [30]. Results of plant competition have traditionally been analyzed by aboveground responses, despite that up to 80% of the plant community biomass may be belowground [31]–[33]. Today it is still unknown how aboveground responses are mirrored belowground [20], and therefore we are missing the contribution of a critical component involved in plant competition [28].

**Figure 1:**
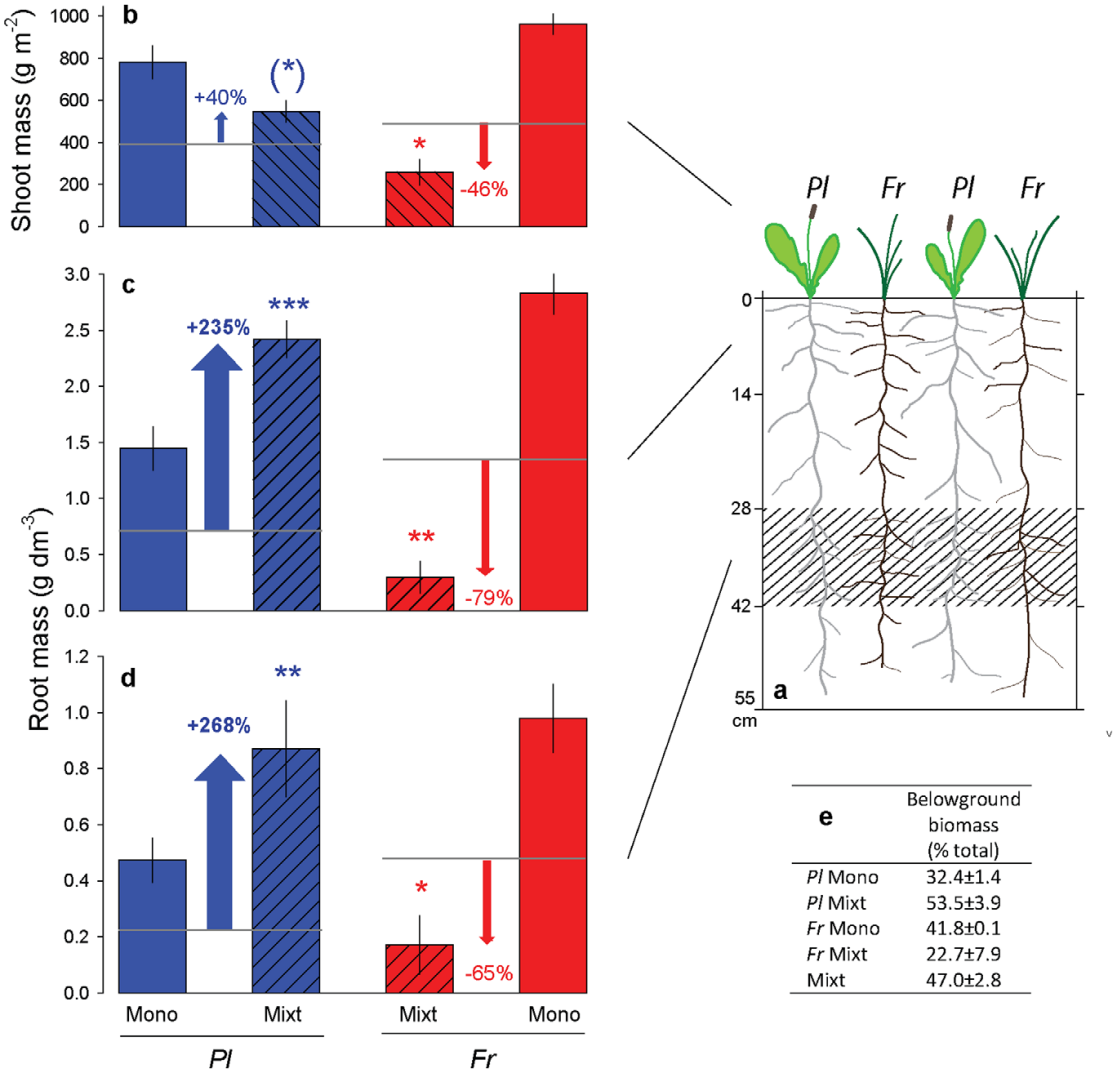
Experimental setup and biomass data. Planting scheme (**a**); shoot (**b**) and root mass in the poor top (**c**) and rich bottom layer (**d**); percentage belowground biomass at harvest (**e**), in *Festuca rubra* (*Fr*) and *Plantago lanceolata* (*Pl*) monocultures and mixtures. Horizontal lines in **b–d** show expected values for mixtures in case of competitive-equivalence (i.e., 50% of monocultures, or a relative yield of 0.5), and arrows depict the percentage deviation. Asterisks show significant differences between observed and expected values after t-tests. Data are means ± SE, N = 3–4. (*) P<0.06; * P<0.05; ** P<0.01; *** P<0.001.

Here, two common West-European grassland perennials, *Plantago lanceolata* L. and *Festuca rubra* L., were led to compete in large containers in a facility specifically designed to study root growth under near-natural conditions for two growing seasons. The 55 cm deep containers in which the communities were grown contained a deep nutrient-rich soil layer (28–42 cm depth; Fig. 1A), with a high concentration of humus-rich soil, to test how these species competed for nutrients placed at depth. We combined monocultures and 50/50 mixtures to test the expectation from resource competition theory that the superior competitor takes resources at the expense of the inferior competitor, leading to a replacement of one species by another [10], [11], [34]. In particular, we expected the species developing the densest roots per unit of soil volume (i.e., *F. rubra)*, quickly taking up the available nutrients in the nutrient-rich soil layer, acquiring a greater fraction of the nutrient supply rate and depleting the soil to the lowest nutrient concentrations (i.e. having the lowest R*), to win the competition [6], [35]. The species with lower root densities (i.e., *P. lanceolata*) would only be expected to win if it would forage more effectively than its competitor for the nutrients in the nutrient-rich layer, or, following game theoretical predictions, if this species would pre-empt belowground space at the expense of its competitor and independent of nutrients. To address belowground responses, minirhizotron images were taken on a monthly basis and root mass in mixtures was determined at final harvest by applying a recent molecular method to quantify root mass of different species in mixed samples [29], [30].

## Methods

### Species and experimental setup

The two species investigated commonly co-occur in hay meadows in central-northern Europe. The research was conducted in the Phytotron of the Radboud University Nijmegen (http://www.ru.nl/phytotron) in containers with separate units of 50(w)×50(l)×70(h) cm each. It is situated under a transparent rain shelter (high-quality commercial greenhouse film, 90% transparency) and open at all sides in order to allow natural weather conditions, except for some wire-netting. Plants were thus grown under near-ambient growth conditions except for watering. Monocultures of *F. rubra* and *P. lanceolata*, or mixtures of both species (1∶1 proportion), were planted in June 2008 in a replacement design. Assignment of the planting treatments occurred randomly to a total of 11 units, resulting in 3–4 replicates. Planted seedlings were raised in the greenhouse for four weeks before transplanting. Seeds from local provenance (forelands of the river Rhine, near Nijmegen, the Netherlands) were first germinated in Petri dishes and then transferred to small pots containing the same background soil until transplant. Thirty-six seedlings (6×6) were then planted in each unit giving a plant density of 144 m^−2^, but only the area of the inner 4×4 plants (32×32 cm) was used for further measurements. Interplant distant was 8 cm but distance from the edge plants to the rim was 5 cm. During the growing season, plants were irrigated 2 L unit^−1^ three times a week with tap water through an automatic irrigation system (PRIVA, de Lier, The Netherlands). In winter, watering was supplied manually once a week.

The bottom of each unit was filled with a five-cm layer of coarse gravel covered with weed cloth. Soil depth from surface to gravel stones was 55 cm, divided in a 14-cm nutrient-rich layer consisting of black soil placed at 28 cm depth, and the remaining of the profile being filled up with a mixture of the same nutrient-rich black soil and nutrient-poor riverine sand (1∶3; v∶v) resulting in a poor sandy background soil. Soil nutrients were measured with an autoanalyzer (Bran+Luebbe, Norderstedt, Germany) after nutrients were extracted by diluting 20 g of freshly mixed soil samples in 50 mL of demineralized water and shaking for 1 h. At the start of the experiment, the nutrient-rich soil contained 26.4±1.5 g kg^−1^ organic matter, and available nutrients were as follow: 256.4±65.1 mg kg^−1^ nitrate (NO_3_^−^), 11.2±2.2 mg kg^−1^ ammonium (NH_4_^+^) and 4.1±0.3 mg kg^−1^ phosphate (PO_4_^−3^), whereas these values were 9.6±0.1, 60.8±0.1, 2.5±0.0 and 1.5±0.3, respectively, for the nutrient-poor background soil.

Each unit had separate drainage at the bottom and holes to insert a minirhizotron tube (6.4 cm inner diameter×50 cm length) horizontally with the top of the tube at 10 cm depth, and two soil suctions cups (Rhizosphere Research Products, Wageningen, The Netherlands) at 7 and 35 cm depth for collecting soil solution for analysis.

### Measurements

In late August 2009, after two growing seasons, standing shoot biomass in the inner area was harvested by clipping 2 cm above soil surface. Root mass density was estimated by soil cores (20 mm diameter, four sub-replicates in each plot) in the inner area down to four soil layers (0–14, 14–28, 28–42, 42–55 cm depth). Distances to surrounding individual plants were equal. Roots per soil increment were collected after carefully rinsing them with tap water. Fresh weight was determined immediately with a microbalance (Sartorius AG, Goettingen, Germany). Up to 100 mg of fresh roots was then stored at −80°C for later molecular analyses. Shoot and root dry weights were determined after drying samples at 70°C for 48 hours. Species abundance belowground in mixtures was quantified only in the top soil layer (0–14 cm) and in the intermediate nutrient-rich layer (28–42 cm), thus processing ≈70% of the total root biomass. On these samples, genomic DNA extracts were subjected separately to quantitative real time polymerase chain reactions (RT-PCR) with primers for non-coding species-specific markers [29]. Analyses were performed on the basis of 100 mg fresh root mass, and recalculated in terms of dry weight as this was highly correlated with the fresh weight (R^2^ = 0.89, P<0.001).

**Figure 2:**
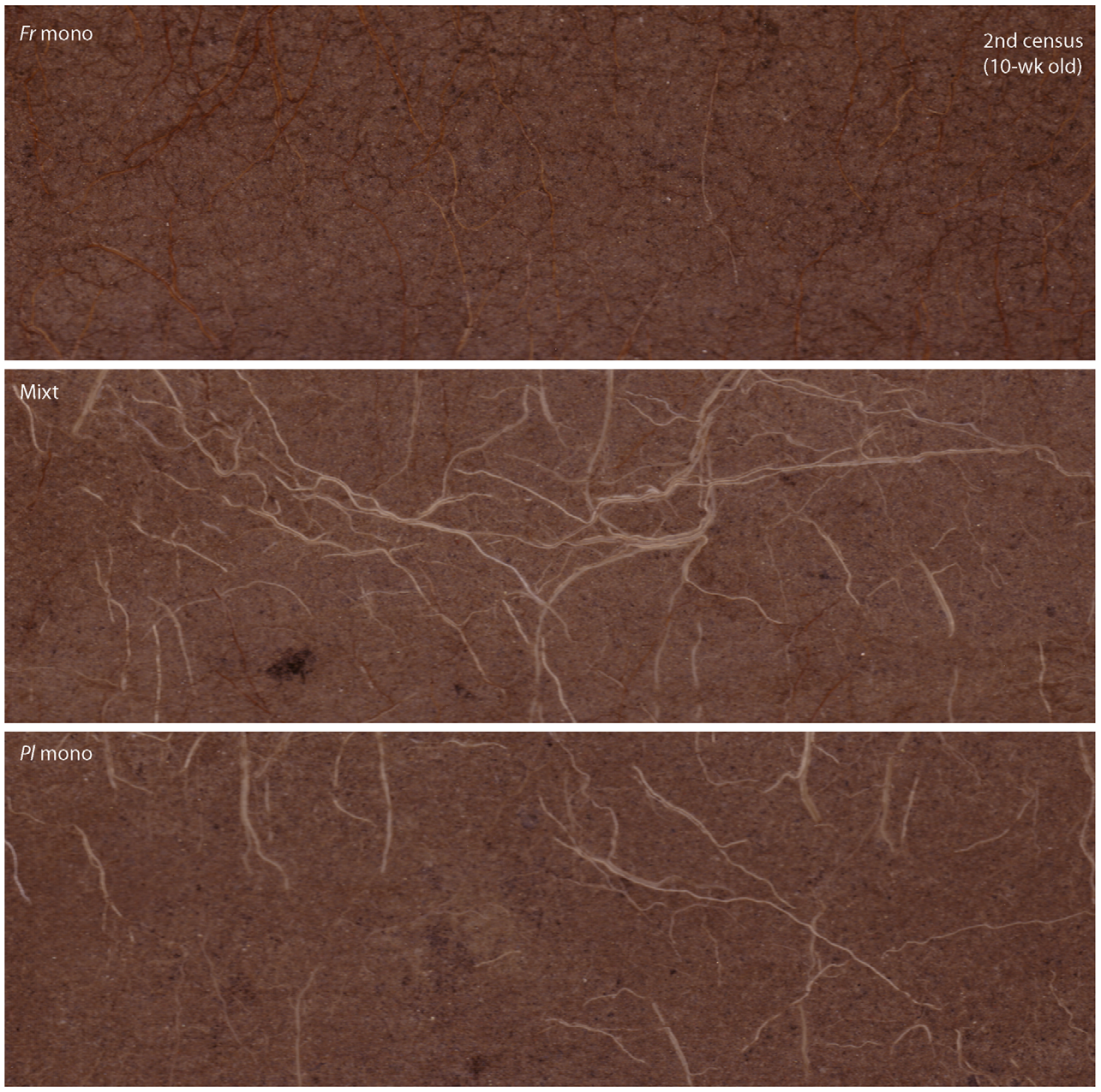
Minirhizotron images at 10 cm depth taken ten weeks after the start of the experiment. Note the abundance of *P. lanceolata* (*Pl*, white roots) and absence of *F. rubra* (*Fr*, brown roots) in mixtures images compared to the respective monocultures.

Root images from minirhizotron tubes at 10 cm depth (21.6×7.0 cm, 300 dpi; CI-600 Root Scanner, CID Inc., Camas, WA, USA) captured the rooting area of four individuals in a row (either of the same species in monocultures, or half of each species in mixtures). Images were taken every 37 days on average, except in winter (Nov–Feb). Roots were digitized and analyzed using the WinRhizoTron V. 2005a software (Regents Inc., Quebec, Canada) for root length production. In mixtures, analyses were separated by species as the different color of newly-formed roots enabled species distinction: dark red to brown roots for *F. rubra*, pale grey to white roots for *P. lanceolata* (Fig. 2).

Soluble nutrients in the soil solution at two depths (7 and 35 cm; poor and nutrient-rich soil, respectively) were monitored every 65 days on average except in winter, by sampling soil water through porous soil suction cups and analyzing the extracted water solution for available nitrate with an autoanalyzer (Bran+Luebbe, Norderstedt, Germany).

### Calculations and data analysis

In our replacement design, where total plant density in the mixture was equal to the plant density used in the monoculture of each component, the competitive ability of the species was given by the Relative Yield [9], [10], [36], calculated as the ratio between the observed yield of a species in mixture and the yield of this same species in monoculture. These calculations find their origin in Lotka-Volterra competition theory [10], which is the central concept in competition and coexistence theory [37], [38]. Relative Yields of 0.5 for both species reflect the situation of competitive equivalence with intraspecific competition equal to interspecific competition. If plants compete for a finite resource, a superior competitor is expected to take a larger proportion of the shared resource resulting in a higher Relative Yield, at the expense of an inferior competitor which will develop a proportionally lower relative yield. Belowground, the root Relative Yields are expected to deviate particularly in the nutrient-rich layer with the superior competitor developing a much larger root mass than the inferior competitor. As root investments will pay-off in nutrient uptake and growth, the root Relative Yields in the nutrient-rich layer are expected to be similar to the Relative Yields aboveground.

As a finite resource is partitioned among competing species, the sum of the relative yields (Relative Yield Total, RYT) is not expected to deviate from unity. RYT>1 or overyielding is only expected in the case of niche differentiation, i.e. when both competitors have access to partly unique resources, as in the case of species with different rooting depths [13]. In such cases intraspecific competition is greater than interspecific competition for both species which is the criterion for species coexistence [38]. Significance of Relative Yields is tested by comparing the observed values with those expected from monocultures representing the null-expectation of competitive equivalence (RY = 0.5), calculated as ½ of the monoculture values.

If the species are involved in a competitive game, one of the species is expected to overinvest in roots (Relative Yield >>0.5) at the expense of the other species (Relative Yield <<0.5). Such investment will take place similarly in the nutrient-poor topsoil as in the nutrient-rich deep soil layer. Depending on the extent of dominance and suppression, RYT may appear larger than 1. As root investments are expected to be altered, Relative Yields belowground will not reflect Relative Yields aboveground.

Root and shoot masses were estimated on the basis of above and below-ground biomass values per area and core soil volume, respectively. To compare whether values of root mass observed in mixtures for each species deviated from the expected values, we ran t-test.

Significance of differences over time in soil solution nutrients were tested by ANCOVA, using diversity of species and soil depth as fixed factors, and days after plantation as covariate. Conventional tests aimed at testing temporal trends (RM-ANOVA and MANOVA) could not be applied because the sphericity assumption was not met. In ANCOVA, differences in the temporal pattern between factors were considered significant when the interaction(s) between factor(s) and ‘days after plantation’ result was significant. When a factor or interaction resulted significant, pair-wise comparisons were performed using the Sidak correction for multiple comparisons.

Differences between expected and observed root length density from minirhizotron images were explored by linear regression, by plotting expected against observed values deviating from the null 1∶1 expectation (i.e., expected = observed). Differences between observed and expected values of root lengths in mixtures on specific dates for each species were tested by t-tests, using Sidak correction for multiple comparisons. All analyses were run with PASW Statistics 18 (SPSS Inc., Chicaco, IL, USA).

## Results

**Figure 3:**
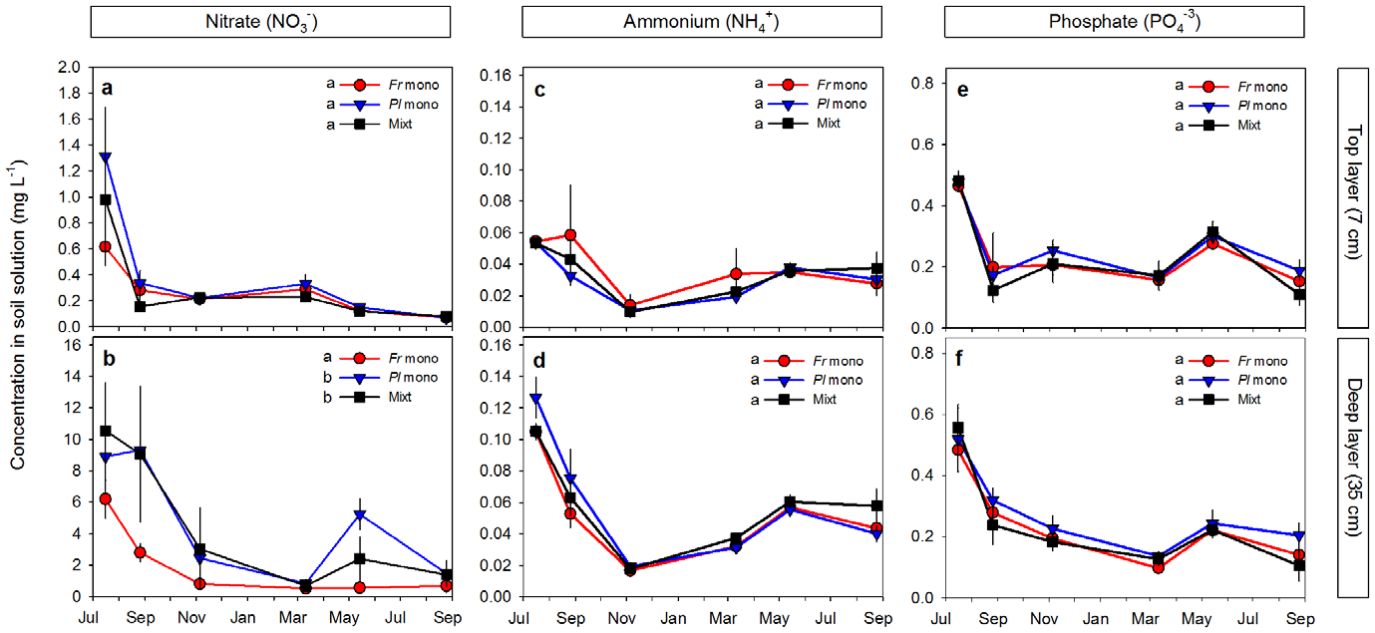
Nutrients dynamics in soil solution over time. Nitrate (**a, b**), ammonium (**c, d**) and phosphate (**e, f**) concentration in *Festuca rubra* and *Plantago lanceolata* monocultures and in mixtures of the two species, at 7 and 35 cm depth. In nitrate, different letters in legends show significant differences between species over time, after ANCOVA_layer x species_. Data are means ± SE, N = 3–4. No significant second and third order interactions involving species and time were detected (Table 1), meaning that similarities/differences between species were consistent all over the experimental period. Soil nutrient concentrations were derived from regular sampling of soil water over the course of the experiment through porous suction cups that had been placed in the soil layers.

*Festuca* was clearly projected as the superior species in nutrient competition: its root length densities in monoculture were 1.4–2.1 times higher than of *Plantago* (Fig. 1, C and D), and soil nutrient solution measurements throughout the study period showed that *Festuca* monocultures more quickly took-up nutrients in the rich soil layer and depleted them to a lower concentration than *Plantago* monocultures (Fig. 3). Nitrate, the most limiting nutrient in these soils and the most differentiating nutrient between the rich and poor layer (Table 1), was much more available in the deeper nutrient-rich layer than in the poor top at the beginning of the experiment, but differences between both layers levelled off as the experiment progressed. In the deep-rich layer, nitrate in *Festuca* monocultures became significantly lower than in *Plantago* monocultures, while in the poor-top layer, availability of nitrate did not differ among communities. Soil nitrate measurements (Fig. 3) further showed that *Festuca* monocultures depleted soil nitrate more rapidly than *Plantago* monocultures from the very beginning of the experiment, although the root densities were still low. If the species behave similarly in mixture than in monoculture, it is to be expected that *Festuca* will more quickly take up available nitrate and develop more root mass at the expense of *Plantago*.

**Table 1:** ANCOVA results for available nutrients in soil solution.

Variable	Source	d.f.	F-value
Nitrate	Species x Depth x Time	2	1.277^ns^
	Species x Depth	2	**4.140** *
	Species x Time	2	1.606^ns^
	Depth x Time	1	**30.285** ***
	Species	2	**5.027** **
	Depth	1	**80.600** ***
	Time	1	**42.499** ***
	Error	120	
Ammonium	Species x Depth x Time	2	0.878^ns^
	Species x Depth	2	1.521^ns^
	Species x Time	2	0.569^ns^
	Depth x Time	1	2.957^ns^
	Species	2	0.322^ns^
	Depth	1	**15.480** ***
	Time	1	**14.675** ***
	Error	118	
Phosphate	Species x Depth x Time	2	0.075^ns^
	Species x Depth	2	0.087^ns^
	Species x Time	2	0.085^ns^
	Depth x Time	1	2.802^ns^
	Species	2	0.055^ns^
	Depth	1	2.266^ns^
	Time	1	**40.007** ***
	Error	117	

Diversity of species (*F. rubra* monoculture, *P. lanceolata* monoculture and mixture of the two species) and soil depth were fixed factors, and time after plantation was a covariate.

* P<0.05;

** P<0.01;

*** P<0.001;

ns P>0.09. Bold shows significant effects.

However, results from the mixtures immediately contrasted with this expectation: *Plantago*, rather than *Festuca*, won the competition belowground. Moreover, *Plantago* did not competitively replace the inferior *Festuca* but strongly overproduced roots and did so both in deep-rich and the poor-top soil layer. This dominance and suppression belowground became established early in the experiment, prior to and not as a result of soil nutrient depletion.

Where *Festuca* was severely reduced in mixtures (72% less root mass than expected from monoculture; t-test obs. *vs.* exp., P = 0.001 top layer, P = 0.038 bottom layer; Fig. 1, C and D), roots of *Plantago* did not simply take the space from which *Festuca* was ousted. Rather, *Plantago* overcompensated and produced on average a massive 252% more root mass in mixtures than expected from monocultures (t-test obs. *vs.* exp., P<0.001 top layer, P = 0.009 bottom layer; Fig. 1, C and D). Root overproduction of *Plantago* in mixtures was so overwhelming that this species had as much (t-test, P = 0.069 bottom layer) or even higher (t-test, P = 0.015 top layer) root biomass in mixtures than in its monocultures, with only half the number of plants.

One basic tenet of resource competition is that a superior competitor takes resources at the expense of an inferior species, resulting in a differentiation of their relative yields from the null-expectation of competitive equivalence (Relative Yield, RY = 0.5) [6]. As both species have access to the same pool of limiting resources, the sum of relative yields (Relative Yield Total, RYT) is not expected to differ from unity [10], [34]. However, with belowground RY of 1.66 and 0.13 for *Plantago* and *Festuca*, respectively, our results significantly deviate from these expectations. A RYT significantly higher than unity can be expected if species have access to different resources, as in species with different rooting depths [13], [18], [19], [39], but this was not the case in our experiment where both species in mixture had similar mean rooting depths (i.e., 7.4±1.0 cm; *F_1,10_* = 0.001, P = 0.973).

Root overproduction of *Plantago* occurred similarly in top (poor soil) and bottom (rich soil) layers, despite their very different nutrient availability (Fig. 3). Overproduction of *Plantago* roots solely in the rich layer could have been interpreted as a nutrient-induced (foraging) response, but the fact that the same degree of overproduction was found in the poor-top layer suggest that the overproduction of *Plantago* was not related to differences in soil nutrients but induced by the presence of the competitor species.

**Figure 4:**
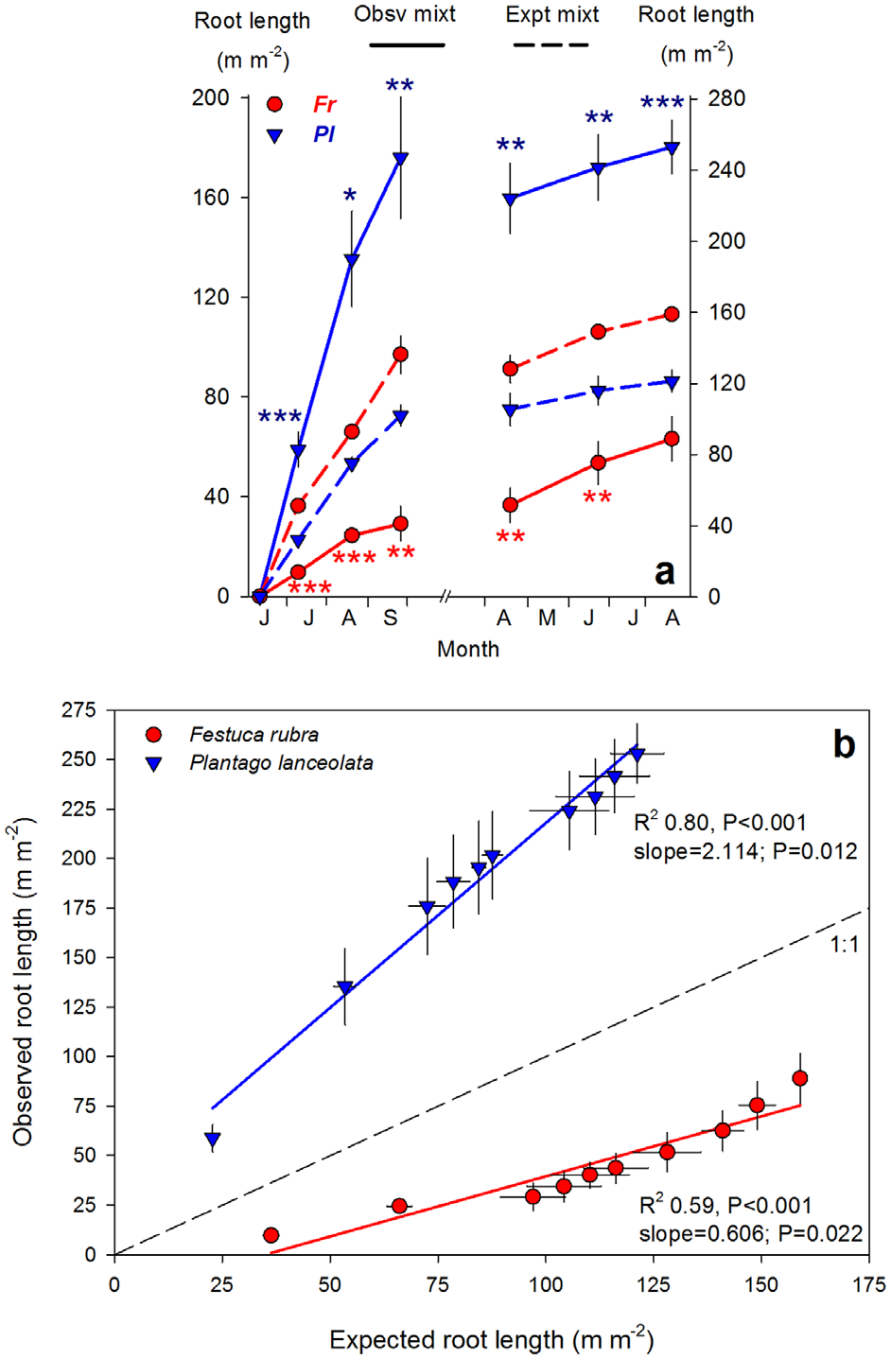
Root growth observed through minirhizotron tubes. (**a**) Root length production over time (m m^−2^ image) of *Festuca rubra* (*Fr*) and *Plantago lanceolata* (*Pl*) in mixtures, obtained from minirhizotron observations at 10 cm depth. Solid lines are for observed values, dashed lines for expected values from monocultures (½ of monocultures). On each date, t-test were run separately to detect significant differences between observed and expected values in each species. *P*-values were then adjusted using the Sidak correction for multiple comparisons. After correction, * P<0.009; ** P<0.002; *** P<0.001. (**b**) Linear regression of expected *versus* observed root length of *Plantago* and *Festuca* in mixtures over the whole experiment, and null expectation expected = observed (1∶1). Significance of deviation of slopes from unity is shown by p-values. Data are means ± SE, N = 3–4.

Non-destructive observations from minirhizotron tubes located in the top layer revealed when root densities of the species started to diverge. Disentangling the roots on the images by color (dark red to brown roots for *Festuca*, pale grey to white roots for *Plantago*, Fig. 2) showed that the root overproduction of *Plantago* was initiated immediately after the start of the experiment (Fig. 4a). Already at first census (four weeks after plantation onwards), *Plantago* produced 3× more root length in mixtures than expected from its monocultures. At this time, root length densities were only 20% of the densities developed after the two growing seasons. Soil nitrate concentrations of the rich layer were still >6-fold higher than later in the experiment and not significantly different between mixtures and *Plantago* monocultures (Fig. 3). These differences in root length were maintained until the end of the experiment. Likewise, immediately after the start of the experiment, *Festuca* produced less root length in mixtures than expected from its monocultures, despite the relatively high soil nutrient concentrations in the mixed soil, and differences remained over the two growing seasons of the experiment (Fig. 4b). Importantly, these results suggest that root overproduction in *Plantago* and suppression in *Festuca* preceded soil nitrate depletion and that they were not the result of higher soil nitrate uptake by *Plantago*.

## Discussion

By analysing the root responses of two common perennials in a straightforward competition experiment, two surprising results were apparent. Firstly, the species projected as the better competitor for nutrients based on the monocultures (*Festuca rubra*) did not win the competition. Soil nutrient and root growth analyses through time revealed a sequence of events that deviated from what may be expected in resource competition. Dominance and suppression were established very early in the experiment, irrespective of soil nutrient availability. Root growth of the superior species (*Plantago lanceolata*) was stimulated in mixtures, and root growth of the inferior species severely reduced, *prior to* nutrient depletion. *Plantago* did not win because it took up a larger proportion of the shared resources after which it was able to develop more roots [1], [22], [40], but because its root growth was immediately stimulated in the presence of *Festuca*. Our results suggest that a species may win competition for nutrients for different reasons than is currently assumed.

Secondly, the massive root overproduction of *Plantago* in mixture and the overyielding belowground (RYT>>1) is inconsistent with niche differentiation as in such case it is to be expected that both species develop Relative Yields larger than 0.5 [13], [34], [38]. In our experiment an RYT>>1 was reached by severe suppression of the inferior species combined with disproportional root growth of the superior species. As disproportional root growth did not only occur in the nutrient-rich layer, it is not a reflection of a better ability to forage for nutrient-rich hotspots of this species. Rather, root overproduction in the presence of another species and independent of nutrients follow game theoretical predictions. Game theory also predicted that investments belowground increase in competition for nutrients, as observed in mixtures relative to monocultures. We discuss the mechanisms and consequences of these two results below.

### Mechanisms of belowground competition

Except that we have been able to rule out a differential response to soil nutrients, we do not know what mechanisms have been driving this strong dominance and suppression early in this competition experiment. There has been a lot of attention in recent years to the effects of species-specific communities of soil pathogens affecting coexistence and production of plant communities [41]–[43]. *Plantago* growth is known to be sensitive to its own conditioned soils due to accumulation of self-harming fungi [44] and root pathogens [45], suggesting the presence of negative plant-soil feedback [46] in monoculture of this species. Mixtures would have been a better environment for *Plantago* roots to grow as self-harming biota would have been diluted. A recent plant–soil feedback experiment showed that *Plantago* monocultures developed 3.2-fold more biomass in the presence of *Festuca* soil biota compared to soil biota of its own, but the reverse was also true: *Festuca* monocultures grew 2.5-fold more biomass on *Plantago* soil than soil of its own [47]. However, it should be noted that soils in the current experiment were not conditioned purposely and, therefore, it is unlikely that species-specific soil biota solely explain the observed root responses. Moreover, differential root growth developed very early in the experiment well before species–specific soil communities were likely built up [48].

Root growth suppression, apparent already at low densities and independent of local soil nutrient concentrations, is reminiscent of allelopathy or chemical interference [49]–[51]. Release of chemical substances may be expected to quickly reduce root growth, as in the case of *Festuca*, but to our knowledge immediate overproduction by the superior species, as observed in *Plantago*, is a phenomenon that has hitherto been unassociated with allelopathy. Some studies have suggested that root exudates can stimulate root growth in response to interspecific neighbors [26], [52]–[55], which would imply that *Plantago* root growth was stimulated by *Festuca*. These phenomena have not been previously described for these species; phytotoxic effects have been attributed only to root exudates of *Festuca rubra* [56].

Facilitation by roots of certain species through the release of organic acids by roots of leguminous species may account for some cases of root growth stimulation in mixtures [57]. However, it seems unlikely that such facilitative mechanism can explain root growth stimulation in our non-leguminous system [20]. As discussed below, there is no sign of facilitation aboveground in our system as overyielding aboveground was not detected.

### Interpreting root overproduction

Consistent with game theoretical predictions [22], *Plantago* won by rapid investment in roots in the presence of *Festuca*, at the same time suppressing *Festuca* and preventing it from acquiring soil resources, resulting in a much larger root investment of *Plantago* than predicted on the basis of classical resource competition. However, from a game theoretical perspective, pertinent questions remain. Firstly, why did *Plantago* win and not *Festuca*? With its higher root densities, fine roots and high nutrient uptake rates, the grass species *Festuca* had a much better starting position to compete for nutrients. For our species pair, well-known traits conferring competitive ability belowground [1], [4], [6] could not predict the competitive outcome. Further research has to unravel the root traits that have predictive power and even then, the outcome may well depend on specific combinations of species and soils.

Secondly, as competition is a process taking place among individuals, why did *Plantago* not overproduce to a similar extent in monoculture? Craine [22] suggested that the best solution for a plant is to alter root allocation in proportion to the root length density of competitors. This exactly seems to have occurred in our experiment: plants with lower root densities (*Plantago*) overproduced roots strongly and won competition from plants with already high root densities (*Festuca*). As root densities in *Plantago* monocultures are lower than in *Festuca* monocultures, as is generally true for forbs versus grasses, a similar competitive game between *Plantago* individuals may have resulted in less overproduction. In other words, making substantially more roots may have paid-off only in competition with *Festuca* individuals, not with other *Plantago* individuals.

### Consequences for plant competition and coexistence

We do not know how common this belowground competitive mechanism is in plant communities. However, if it is widespread it may have easily gone unnoticed in many competition experiments. The reason is that, aboveground, competitive relationships among our species appeared to conform to the resource competition model. Similar to numerous other experiments with only aboveground information (e.g., [9]; see refs there), Relative Yields of 0.70 and 0.27 for *Plantago* and *Festuca*, respectively, would project *Plantago* as the winner replacing *Festuca* in resource competition (Fig. 1B), further confirmed by an aboveground RYT similar to unity (0.97). Due to inherent difficulties in quantifying the roots of different species, our experiment is one of the first to compare competitive interactions aboveground with those belowground. Doing so revealed that apparently classical competitive relationships aboveground were combined by unexpected responses belowground.

If our results for these two common plant species are representative for a wider group of plants, the implications for long-term competitive superiority and coexistence may be profound. Competitive games are predicted to generate a “Tragedy of the Commons” where plants invest more to the acquisition of a limiting recourse than is optimal in the absence of competition [22]. Likewise, *Plantago* individuals invested 65% more biomass in their roots in mixtures than in monoculture (percentage total biomass increased from 32.4 to 53.5; Fig. 1E). If *Plantago* roots had not overproduced but only replaced the roots of *Festuca* in mixture (belowground RY 0.87 rather than 1.66), this increase in root investment would only have been 16% (percentage total biomass increase from 32.4 to 37.7). Although the investment pays-off in terms of immediate competitive gain, such major root investment may compromise biomass production in the long run, reminiscent of a Tragedy of the Commons. Interestingly, in a two-species *Plantago-Festuca* mixture within a long-term biodiversity experiment [58], *Plantago* initially dominated the mixture aboveground as in our experiment. But over the 11 years of study *Plantago* never outcompeted *Festuca* and after eight years *Festuca* even gained in abundance (J. van Ruijven, pers. comm.). This trajectory suggests that the overinvestment of *Plantago* in roots may have compromised its competitive ability in the long run.

Consistent with our results, there are indications from biodiversity studies that root mass is increased in species mixtures [53], [57], [59] and that this higher root biomass may already develop prior to positive effects of biodiversity on aboveground production [59]. Moreover, evidence is increasing that interactions in multi-species communities are driven by species-specific soil biota giving opportunities for local coexistence [41]–[43], [47], [60], [61], whereas opportunities for niche partitioning for nutrients seem to be limited [28], [62]. Future work should demonstrate to what extent the root responses seen in our experiment also play a role in more diverse plant communities.
